# Supplementary material for: Ladder-shaped microfluidic system for rapid antibiotic susceptibility testing
Source: Commun Eng. 2023 Apr 3;2:15. doi: 10.1038/s44172-023-00064-5 (PMC10956002; doi:10.1038/s44172-023-00064-5)
Supplement: Supplementary file 2 — Supplementary Information [file 44172_2023_64_MOESM2_ESM.pdf]

Supplementary Figures and Tables

Ladder-shaped microfluidic system for rapid antibiotic susceptibility testing

Ann V. Nguyen[1], Mohammad Yaghoobi[1], Morteza Azizi[1], Maryam Davaritouchaee[1], Kenneth W. Simpson[2], Alireza Abbaspourrad[1*]

[1]*Department of Food Science, College of Agricultural and Life Sciences, Cornell University, Stocking Hall, Ithaca, NY, 14853 USA*

[2]*Department of Clinical Sciences, College of Veterinary Medicine, Cornell University, 602 Tower Rd., Ithaca, NY, 14853 USA*

***Corresponding author:** Alireza Abbaspourrad **Email:** Alireza@cornell.edu Tel.: (607) 255-2923

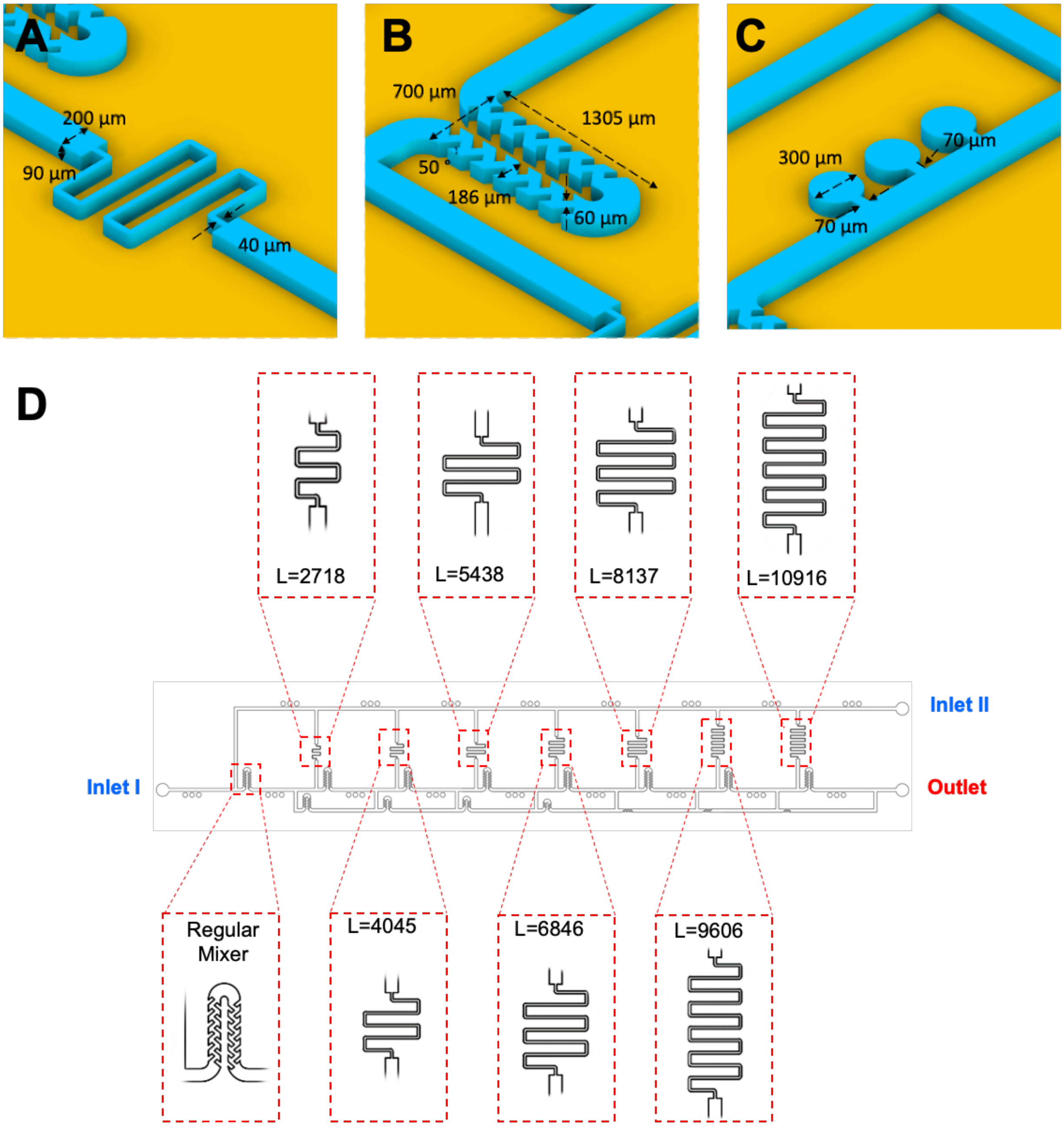

Figure S1. A-C) Detailed dimensions of the ladder shape microfluidic system. D) The length (L, in µm) of the hydraulic constrictions on the side channels.

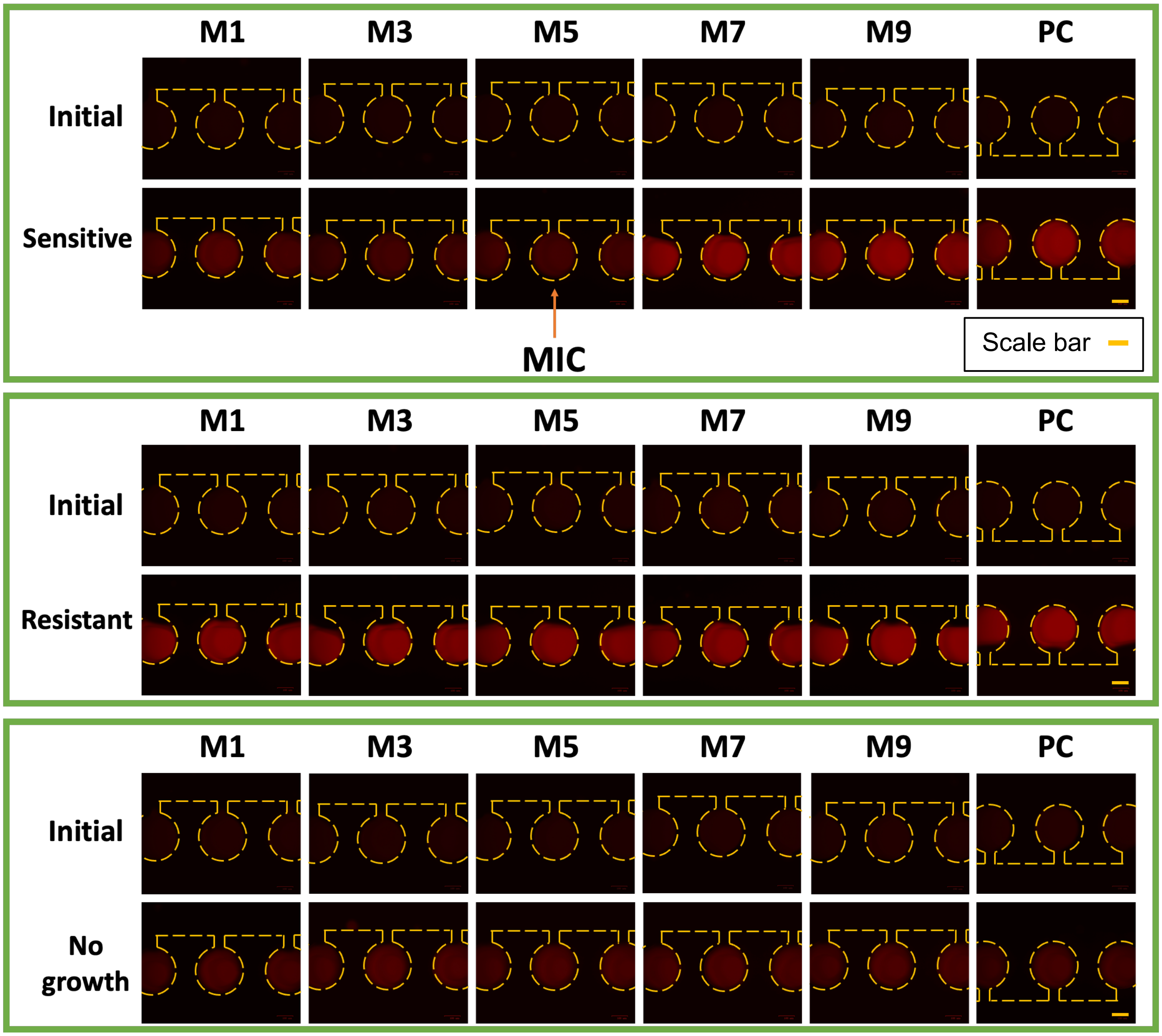

Figure S2. The fluorescent intensity patterns of resazurin in the microchambers at initial time and after 4 h illustrating three possible scenarios: a minimum inhibitory concentration (MIC), antibiotic resistance, and invalid result due to no growth. Scale bar = 100 μm.

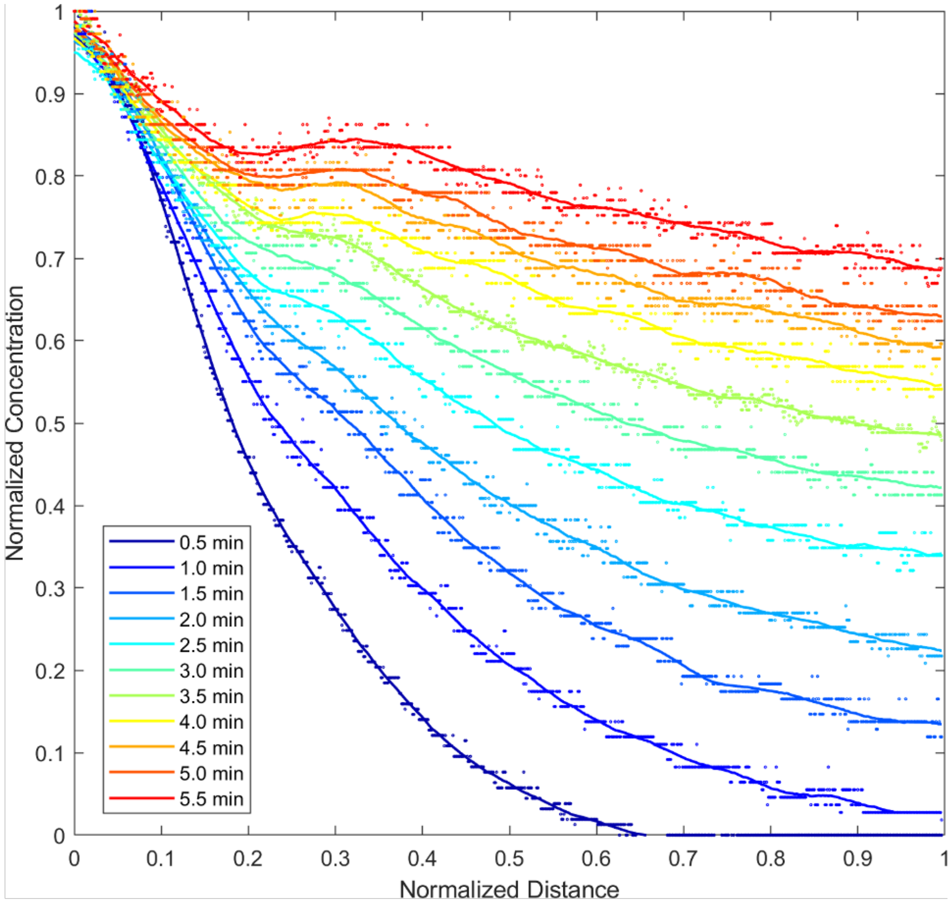

Figure S3. Relative concentration profiles showing the kinetics of resazurin diffusion into a microchamber over time. Raw data used for calculation of the area under the curve of the concentration profile at each time point.

Table S1. Linear correlation between the concentration profiles from computer simulation, of resazurin, fluorescein, and Calcein formed in the ladder microfluidic system, and theoretical 2-fold dilution concentration profile.

Resazurin	$Y= 1.0243x + 0.4781; R^2= 0.9997$
Fluorescein	$Y= 1.0029x – 0.3674; R^2= 0.9982$
Calcein	$Y= 1.0427x + 0.6560; R^2= 0.9984$
Simulated	$Y= 1.0121x + 0.6331; R^2= 0.9983$

Table S2. Summary of the antibiotics used in the study. Source: NCI Thesaurus, DrugBank Online (https://ncithesaurus.nci.nih.gov/ncitbrowser/)

Antibiotic name	Chemical Structure	Class	Mechanism of Action	Targeted Bacteria	Main Route of Administration
Enrofloxacin	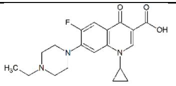	Fluoroquinolone	Inhibition of nucleic acid synthesis	*Staphylococcus, Escherichia coli, Proteus, Klebsiella*, *Pasteurella, Pseudomonas*[1]	oral or subcutaneous
Ceftiofur	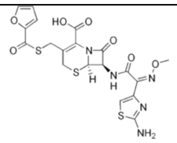	Beta-lactam, third-generation cephalosporin	Inhibition of cell wall synthesis	Broad spectrum	subcutaneous
Tetracycline	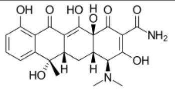	Naphthacene	Inhibition of protein synthesis	Broad spectrum	oral
Cefalexin/ cephalexin	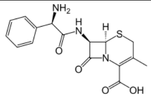	Beta-lactam, first-generation cephalosporin	Inhibition of cell wall synthesis	Gram-positive bacteria	oral
Ampicillin	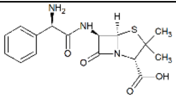	Beta-lactam penicillin	Inhibition of cell wall synthesis	Broad spectrum	oral or subcutaneous
Amoxicillin	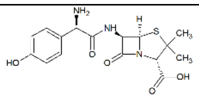	Aminopenicillin	Inhibition of cell wall synthesis	Broad spectrum	oral or subcutaneous
Trimethoprim	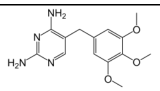	Dihydrofolate Reductase Inhibitor	Inhibition of nucleic acid synthesis	Gram-negative and coagulase-negative *Staphylo coccus* spp	oral
Sulfamethoxazole	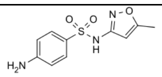	Dihydrofolate Reductase Inhibitor	Inhibition of nucleic acid synthesis	Broad spectrum	oral

Supplementary Reference

(1) Auer, J. A.; Stick, J. A. *Equine Surgery-E-Book*; Elsevier Health Sciences, 2018.
